# Peptide array based discovery of synthetic antimicrobial peptides

**DOI:** 10.3389/fmicb.2013.00402

**Published:** 2013-12-25

**Authors:** Chris W. Diehnelt

**Affiliations:** Center for Innovations in Medicine, The Biodesign Institute at Arizona State UniversityTempe, AZ, USA

**Keywords:** peptide arrays, antimicrobial peptides, targeted antibiotic, antimicrobial resistance, drug discovery

## Antimicrobial peptides as a means to combat antibiotic resistance

The rise of antibiotic resistance has emphasized the shortcomings in antibiotic drug development (Boucher et al., 2013). The move from biological based discovery methods to chemical approaches to identify candidates has left the antibiotic pipeline painfully dry (Lewis, 2013). The paucity of compounds that are effective against antibiotic resistant pathogens has led to great interest in antimicrobial peptides (AMPs) as potential solutions to the rise of resistant organisms (Hancock and Sahl, 2006; Fox, 2013). AMPs are short (5–50 amino acid) peptides that are produced by virtually all organisms as part of an innate immune system. There are 2,398 AMPs that have been reported (Antimicrobial Peptide Database—September 2013) and over 80% are cationic AMPs (CAMPs). Most positively charged AMPs interact with anionic bacterial membranes (Schmidtchen and Malmsten, 2013) which leads to a rapid breakdown in membrane function and subsequent cell death (Wimley, 2010). It is this mechanism of action that is of interest as it should be difficult for bacteria to develop resistance against lethal concentrations of CAMPs.

However, many AMPs have poor drug-like properties and questions remain about that their ultimate utility as antibiotics (Brogden and Brogden, 2011). Great strides have been made in improving the protease stability; pharmacokinetics and therapeutic profile of peptide drugs and these methods have been used to improve the drug-like properties of AMPs. Despite the significant developments that have been made to advance AMPs through the clinical pipeline there has yet to be an approved AMP therapeutic (Vila-Farres et al., 2012). Clearly there is an ongoing need for additional AMP candidates as a tool in the fight against antibiotic resistant bacteria.

## Discovery of synthetic AMPs

Many groups are turning to non-natural sources to discover the next generation of AMPs. These efforts are focused on computational design of AMPs or by screening large libraries of peptides for new candidates. There have been significant advances in computational design of AMPs and progress continues in this field, illustrated by the recent work of Deslouches et al. (2013). However, these studies are guided by rules learned from natural AMPs and could be limiting in terms of designing peptides that function like natural AMPs with all of their inherent strengths and weaknesses. Other groups have used peptide discovery systems to screen large libraries of peptides with the aim of identifying synthetic AMPs and potentially novel classes of AMPs. Display technologies, such as phage display, are capable of producing large libraries of peptides (~10^7^ peptides) that can be used to discover AMPs (Huang et al., 2012). However, display techniques can be difficult to adapt to whole bacteria screening and require multiple rounds of selection to identify peptides with activities similar to natural AMPs.

A promising approach to discover antibacterial candidates is to screen a target bacterium against a peptide library arrayed on a solid surface. This approach uses two different types of peptide libraries: *in situ* synthesized peptide arrays and libraries of peptides prepared as spotted peptide microarrays. Seminal work in the use of *in situ* peptide microarrays for AMP development was demonstrated for small libraries of variants of natural AMPs (Hilpert et al., 2005, 2007, 2009; Hilpert and Hancock, 2007). In this method, hundreds to thousands of peptides are synthesized on a nitrocellulose membrane, then chemically cleaved into micro well-plates where there are then tested for activity. In contrast to this approach, spotted peptide microarrays are prepared by the synthesis of thousands to tens of thousands of peptides, which are printed on glass slides using standard microarray printing technology. Peptide microarrays have been used for ligand discovery by many groups and assays have been developed to screen whole cells against immobilized ligands (Papp et al., 2012).

## Discovery of antibacterial peptides directly on peptide microarrays

**Figure 1:**
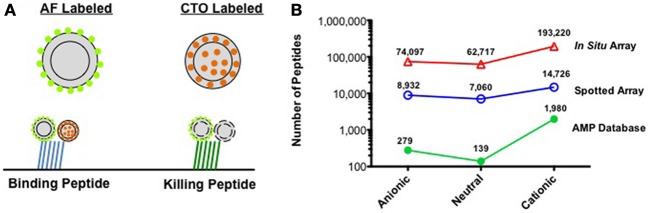
**(A)** Activity based bacterial screening to identify peptides that bind and those that kill a specific pathogen. AF, AlexaFluor-555; CTO, Cell-Tracker Orange. **(B)** Number of peptides based on net charge for AMPs described in the AMP Database, the 30,000 peptides used in spotted peptide arrays, and the 330,000 peptides used in the first generation *in situ* peptide array produced in our laboratory.

We have recently introduced an activity based assay that enables the selection of peptides with antibacterial activity directly on peptide microarrays (Domenyuk et al., 2013). In this assay, the target bacteria is labeled with an internal dye that fluoresces while the cell is metabolically active (e.g., Cell-Tracker Orange) and the exterior of the bacteria (outer membrane for Gram-negative or peptidoglycan layer for Gram-positive bacteria) is labeled with an amine-reactive dye, such as AlexaFluor-555 (Figure 1A). Fluorescently labeled bacteria are then screened against a spotted peptide microarray of 10,000, 20 amino acid long peptides to identify peptides that bind the bacteria and those that kill the bacteria. Peptides that bind but do not kill the bacteria produce two fluorescent colors, while those that bind the bacteria and disrupt the membrane produce one signal. Using this system, we were able to identify peptides that inhibited growth of both Gram-positive and Gram-negative bacteria with minimum inhibitory concentrations (MIC's) in the 20 μM range.

This approach has several important advantages as a source of antibacterial peptides. First, the system can be used to screen a wide variety of bacteria. Laboratory strains or clinical isolates can be easily labeled and do not require genetic modification to express a fluorescent or colorimetric indicator. The labeling procedures are robust and effective for Gram-positive and Gram-negative bacteria. Second, the method is rapid; bacteria can be labeled, screened, and analyzed in the same day. Additionally, the convenience of solid phase synthesis enables the incorporation of non-natural amino acids, such as D-amino acids or β-amino acids, into peptide libraries, enabling the direct screening of protease stabilized peptides. The *in vitro* assay format is very flexible in terms of the screening conditions, buffers, sera or media that can be used. Finally, peptide libraries can be designed without the inherent biases present in natural AMPs, potentially enabling the discovery of active peptides that function with novel mechanisms of action.

## Future directions for peptide array based discovery—selection of peptides that specifically target AMP resistant bacteria

In our opinion, the future of array based AMP discovery lays in the selective targeting of antibiotic and AMP resistant bacteria over normal flora. This could be possible due to the convergence of several parallel avenues of technical and scientific development. Recently, robust methods for the *in situ* synthesis of peptide microarrays with medium densities, >10^3^–10^4^ peptides (Loeffler et al., 2012; Price et al., 2012), and those with much higher densities, 10^5^–10^6^ peptides per array (Legutki, submitted) have been reported. This significantly expands the peptide sequence space that can be explored in a single experiment. This could be an especially important development in the search for peptides that are active against either intrinsically AMP resistant bacteria, such as *Burkholderia cepacia* complex (Loutet and Valvano, 2011), or those that acquire AMP resistance after treatment with CAMPs (Anaya-López et al., 2013; Fernández et al., 2013; Napier et al., 2013; Pelletier et al., 2013; Shireen et al., 2013). Acquired AMP resistance generally involves membrane modifications that increase the charge of the surface of the bacteria that prevents binding of CAMPs to the cell surface. This potentially is a troubling development as the vast majority of AMPs (>80%) discovered to date are cationic and there is some evidence that acquired resistance for one CAMP can extend to host AMPs (Napier et al., 2013). The latter report argues for the selection of AMPs that are not related to natural ones. For these pathogens, it is possible that neutrally charged or anionic AMPs will be effective against AMP resistant bacteria that have membranes modified with cationic groups.

It is here that the recent advances in *in situ* peptide array synthesis could become important. As peptide libraries can be designed with a more even distribution of charged peptides, large numbers of neutral and anionic peptides can be screened against a resistant pathogen. This is illustrated in Figure 1B, where the number of anionic, neutral, and cationic peptides is plotted for: the peptides in the AMP database, the 30,000 peptides we have used in our spotted peptide libraries, and the 330,000 peptides of our recent high-density *in situ* peptide arrays. As can be seen, peptide arrays offer the opportunity to screen thousands to over 130,000 neutral and anionic peptides in a single experiment. It is possible that by screening both the AMP resistant and sensitive phenotype of a pathogen against this array, one could identify reduced charge peptides that inhibit the resistant phenotype with little effect on the sensitive phenotype. It is also possible that a peptide that selectively targets the AMP resistant bacteria would have a much narrower spectrum of activity toward normal flora. The selective targeting of the pathogen should help limit the spread of resistance to other species in the microbiome and maintain normal flora. It is likely that as the understanding of the host microbiome increases, the importance of targeted therapeutics will be even more evident. The use of molecular methods to quickly identify bacteria from clinical specimens is rapidly being adopted and should enable physicians to match a targeted antibiotic with the correct pathogen. Advances in peptide array discovery assays could provide a system to develop pathogen-specific antibiotics (Casadevall, 2009; Lemon et al., 2012) and lead to the discovery of the first generation of targeted antibiotics.
